# Instrumented Functional Reach Test Differentiates Individuals at High Risk for Parkinson’s Disease from Controls

**DOI:** 10.3389/fnagi.2014.00286

**Published:** 2014-10-24

**Authors:** Sandra E. Hasmann, Daniela Berg, Markus A. Hobert, David Weiss, Ulrich Lindemann, Johannes Streffer, Inga Liepelt-Scarfone, Walter Maetzler

**Affiliations:** ^1^Department of Neurodegenerative Diseases, Center for Neurology, Hertie Institute for Clinical Brain Research, University of Tübingen, Tübingen, Germany; ^2^German Center for Neurodegenerative Diseases (DZNE), Tübingen, Germany; ^3^Department of Clinical Gerontology and Rehabilitation, Robert-Bosch-Hospital, Stuttgart, Germany; ^4^Janssen Research and Development, Janssen-Pharmaceutical Companies of Johnson and Johnson, Beerse, Belgium

**Keywords:** balance, limit of stability, neurodegeneration, prodromal Parkinson’s disease, sway

## Abstract

The functional reach (FR) test as a complex measure of balance including limits of stability has been proven to differentiate between patients with Parkinson’s disease (PD) and controls (CO). Recently, it has been shown that the instrumentation of the FR (iFR) with a wearable sensor may increase this diagnostic accuracy. This cross-sectional study aimed at investigating whether the iFR has the potential to differentiate individuals with high risk for PD (HRPD) from CO, as the delineation of such individuals would allow for, e.g., early neuromodulation. Thirteen PD patients, 13 CO, and 31 HRPD were investigated. HRPD was defined by presence of an enlarged area of hyperechogenicity in the mesencephalon on transcranial sonography and either one motor sign or two risk and prodromal markers of PD. All participants were asked to reach with their right arm forward as far as possible and hold this position for 10 s. During this period, sway parameters were assessed with an accelerometer (Dynaport, McRoberts) worn at the lower back. Extracted parameters that differed significantly between PD patients and CO in our cohort [FR distance (shorter in PD), anterior–posterior and mediolateral acceleration (both lower in PD)] as well as JERK, which has been shown to differentiate HRPD from CO and PD in a previous study, were included in a model, which was then used to differentiate HRPD from CO. The model yielded an area under the curve of 0.77, with a specificity of 85%, and a sensitivity of 74%. These results suggest that the iFR can contribute to an assessment panel focusing on the definition of HRPD individuals.

## Introduction

There is a great need for biomarkers in the prodromal phase of Parkinson’s disease (PD) because valid definitions of this phase, and its progression would open entirely new opportunities for treatment and even prevention of neurodegeneration (Postuma et al., 2012a; Berg and Bandmann, 2013; Wang et al., 2013; Lerche et al., 2014). Motor parameters seem to be particularly promising for this purpose as subtle motor changes in individuals at high risk for PD (HRPD) may occur several years before clinical diagnosis can be made. This has been shown for distal (Gaenslen et al., 2011; Postuma et al., 2012b) as well as for axial motor symptoms such as gait and balance (Mirelman et al., 2011; Maetzler and Hausdorff, 2012; Maetzler et al., 2012). Particularly, the latter studies (Mirelman et al., 2011; Maetzler et al., 2012) indicated that challenging test situations may be more effective in delineating subtle motor deficits in this prodromal phase, than do non-challenging test situations. More specifically, we could recently demonstrate that HRPD individuals show, under challenging static balance conditions, a higher variability of trunk acceleration and a lower smoothness of sway (indicated by higher JERKs) in both anterior–posterior (AP) and mediolateral (ML) direction, compared to both controls (CO) and PD.

The limit of stability is a dimension of the postural control system, which reflects the maximum displacement of the body’s center of mass over a fixed base of support of the feet without losing balance (Horak et al., 2005). The functional reach (FR) test enables to determine this limit of stability in the AP direction, by reaching forward during quiet standing (Duncan et al., 1990). A good and practical definition of the FR is the greatest distance in any direction a person can reach out from a midline verticale position without falling or stepping. Thus, the FR is an operationalization of “the self-perceived limits of stability” (Mancini et al., 2008). The FR has been shown to differentiate older fallers from non-fallers (Duncan et al., 1992; Huang et al., 1998; Almeida et al., 2012), and the FR distance is a useful outcome parameter for fall prevention programs and progressive strength training (Sousa and Sampaio, 2005; Lin et al., 2007). Importantly, the test has also been shown to discriminate between PD patients and CO. One study (Smithson et al., 1998) found that PD patients have a shorter FR distance (~4 cm) than CO, which has been confirmed by another study (Mancini et al., 2008). Recently, first data about an instrumented FR (iFR) using a wearable sensor during the task have been presented (Cattabriga et al., 2013). The data indicate that the approach is feasible, and may improve diagnostic accuracy of PD.

Comparing HRPD individuals with PD patients has its weaknesses, in particular, in experiments that test dysfunction and compensation mechanisms in parallel, and these mechanisms are difficult to disentangle. A particular strength of the analysis of an iFR can be the consideration of two components: distance (how far someone can reach) and behavior (how does someone “behave” at her/his self-perceived limit). The further a study participant reaches due to motivation issues (sensorimotor integration), the better is the distance value but the worse are the sway parameters, and vice versa. Moreover, we included the parameters shown previously to be different in HRPD and CO (Maetzler et al., 2012) in our model, especially because previous data suggests a *U*-shaped progress of some parameters from CO over HRPD, to PD. A *U*-shaped process can contain compensatory and/or adaptation mechanism, as well as hidden pathophysiological aspects.

To our knowledge, there is no study available yet on changes of stability limits in prodromal PD. As already stated, subtle motor changes can be detected with quantitative assessment tools before the clinical diagnosis can be made (Yang et al., 2008; Mirelman et al., 2011; Maetzler and Hausdorff, 2012; Maetzler et al., 2012). Limits of stability are reduced in PD (Rossi et al., 2009; Menant et al., 2011) and can be found even in early untreated disease stages (Mancini et al., 2012). Thus, we were interested whether we could detect differences between PD and CO in challenging limits of stability paradigm (Duncan et al., 1990; Kamata et al., 2007).

Moreover, based on these assumptions and our previous results we were interested in the potential of the iFR to differentiate between HRPD individuals and CO.

## Materials and Methods

### Ethics

The ethical committee of the Medical Faculty of Tuebingen approved the study protocol and written informed consent was sought from all participants (Liepelt-Scarfone et al., 2013).

### Individuals

In this cross-sectional study, 13 PD patients, 13 CO, and 31 HRPD individuals were included. The study presented here is part of the observational PMMP study on HRPD individuals, for details we refer to Maetzler et al. (2012), Liepelt-Scarfone et al. (2013), Louter et al. (2014). In brief, PMMP stands for “progression markers in the premotor phase” of PD, which is a prospective longitudinal 2-year study. The aim of the study is to monitor the progression of the disease until the development of (subtle) motor changes in older adults with risk factors for PD. All HRPD had an enlarged area of hyperechogenicity of the substantia nigra on transcranial sonography (>0.19 cm^2^ on at least one side). The enlarged area of hyperechogenicity in the mesencephalon is one of the most relevant risk factors for future PD in individuals older than 50 years (Berg et al., 2011). Additionally, either one cardinal motor sign of PD (slight bradykinesia, rigidity, tremor, postural instability) assessed by the unified Parkinson disease rating scale motor part (UPDRS-III), or two of a set of well-established risk and prodromal markers: positive family history, one-sided reduced arm swing, history of depression, and hyposmia (<75% correct answers in the identification test of the Sniffin’ Sticks) have to be present. PD diagnosis was excluded for HRPD and CO by clinical investigation. We decided to include individuals with a combination of markers as the accumulation of risk/prodromal factors in an individual increases the risk of getting PD at least linearly (Liepelt et al., 2011; Ross et al., 2012; Siderowf et al., 2012). Demographics and clinical characteristics are illustrated for the three groups (Table 1).

**Table 1:** **Demographics and clinical parameters**.

	PD (*N* = 13)	Co (*N* = 13)	HRPD (*N* = 31)	*p*-value
Age (years)	65.0 (9.4)	63.9 (7.3)	62.6 (5.0)	0.53
Male sex (%)	8 (62)	7 (54)	23 (74)	0.38
Height (m)	1.73 (0.08)	1.71 (0.09)	1.74 (0.06)	0.64
Weight (kg)	77 (11)	72 (6)	78 (12)	0.23
BMI (kg/m^2^)	25.6 (2.8)	24.6 (1.9)	25.8 (3.2)	0.44
MMSE (0–30)	29.3 (0.9)	29.7 (0.5)	29.1 (0.8)	0.10
BDI (0–63)	9.6 (8.3)	2.9 (3.6)*	5.7 (4.8)	0.01
UPDRS-III (0–129)	26.8 (11.0)	0.2 (0.6)*	3.0 (3.0)*,^#^	<0.0001
SN+ (cm^2^)	0.24 (0.04)	0.12 (0.03)*	0.26 (0.05)*,^#^	<0.0001
Age at disease onset (years)	60.5 (8.9)			
Disease duration (years)	4.5 (2.8)			

*Data are presented with the mean and SD, or with frequency. *p*-values were assessed using ANOVA with *post hoc* Student’s *t*-test or with the Pearson Chi square test*.

*BDI, Beck’s depression inventory; BMI, body mass index; Co, controls; HRPD, individuals at high risk for future Parkinson’s disease (PD); MMSE, mini-mental state examination; SN+, hyperechogenicity of the mesencephalic region including the substantia nigra; UPDRS-III, motor part of the unified Parkinson disease rating scale*.

*p < 0.017 compared to PD.

*^#^*p* < 0.017 compared to controls (Co)*.

### Functional reach test and extraction of quantitative data

**Figure 1:**
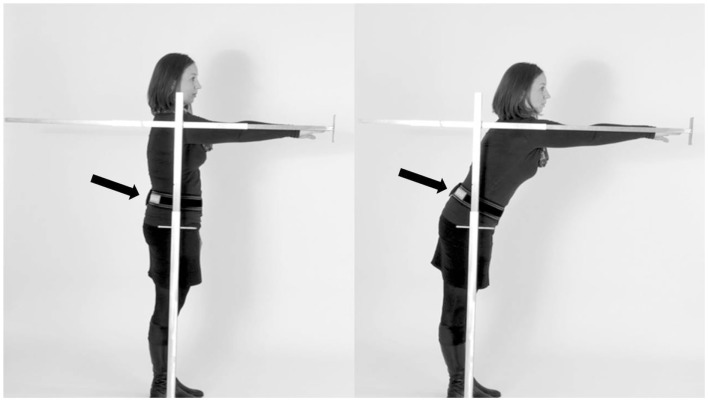
**Performance of the instrumented functional reach test (SEH)**. Participants were asked to stand upright **(A)**, reach forward as far as possible by pushing the rod, and then to hold the position for 10 s **(B)**. The sensor was worn at the lower back (arrow).

All participants stood upright in narrow stance, the right arm reaching out without bending forward. The start and end positions were assessed with a metal rod, which was movable and included a tapeline. The participants were instructed to touch a small plate at the end of the rod with their right fingertip, to push the plate forward as far as possible, and then to hold this position for 10 s without performing a compensation step (Figure 1). Then the participants moved back to the initial position. The FR distance was measured in centimeters. During the assessment, all study participants wore an accelerometer (Dynaport Hybrid^®^, McRoberts, The Hague, The Netherlands) at the lower back.

Only sensor data from the maximal forward reach phase – in which individuals had to hold the determined position – were extracted and analyzed. We did not quantify the transition phase, which has previously also been shown to be associated with fall risk (Cattabriga et al., 2013).

### Statistics

Statistical analyses were performed with JMP 10.0, SAS. Demographic and clinical data are presented with mean and SD, or with frequency. *p*-Values were calculated using Student’s *t*-test, ANOVA, or Pearson’s Chi square test (Table 1). Quantitative FR parameters were compared between PD patients and CO using Student’s *t*-test after testing for normal distribution. Non-normally distributed parameters (JERK AP and ML) were log-transformed before analysis. Parameters that reached a *p*-value below 0.05 were included in a *logistic* regression model. Sensitivity and specificity in differentiating HRPD to CO was calculated by ROC-analysis. The additional value of inclusion of parameters was confirmed by an increase of *r*^2^ (Table 3). Second, another regression model was calculated, which additionally included JERK in the AP and ML direction. These parameters have been shown to differentiate HRPD from CO and PD in a *U*-shaped manner (Maetzler et al., 2012), and may thus be overlooked by the above explorative model, which assumes linear changes of parameters in the disease course.

## Results

### Characteristics of the cohorts

Differences in age, gender, weight, height, and MMSE score did not reach significance among the investigated cohorts (Table 1). PD patients had significantly higher UPDRS and BDI (*p* < 0.017) scores, indicating more severe motor problems and depressive symptoms, than both CO and HRPD. Probably due to the inclusion criteria (see above), CO had lower UPDRS values than HRPD individuals. Both PD and HRPD individuals had comparable echogenicity of the substantia nigra (SN+) values, which were both, as a mean, significantly larger than those of CO (*p* < 0.017, Table 1).

### Quantitative FR analysis between PD patients and controls

**Figure 2:**
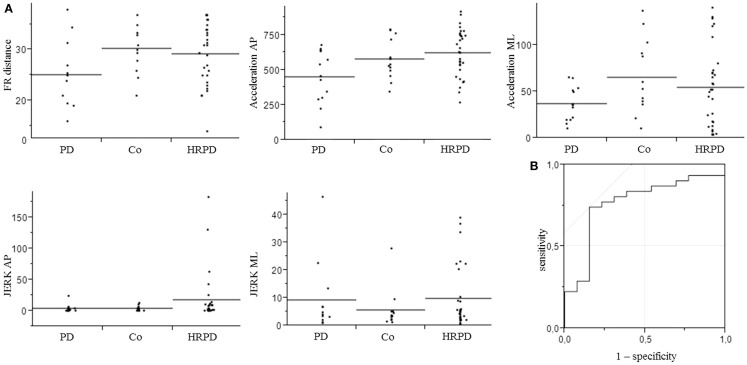
**Parameters included in the model for the differentiation of controls from individuals with high risk for Parkinson’s disease (A), which yielded an area under the curve (AUC) of 0.77, with a specificity of 85%, and a sensitivity of 74% (B)**. AP, anterior–posterior.

All individuals were able to perform the trial correctly within the first trial. PD patients differed from CO in the following parameters: FR distance (*p* = 0.03), AP acceleration (*p* = 0.04), and ML acceleration (*p* = 0.03, Figure 2). No significant differences could be detected for the following parameters: area of sway, velocity (AP and ML), JERK (AP and ML), and mean power frequency (*p* > 0.05, Table 2).

**Table 2:** **Quantitative functional reach (FR) parameters of patients with Parkinson’s disease (PD), controls (Co), and individuals with high risk for PD (HRPD)**.

	PD (*N* = 13)	Co (*N* = 13)	*p*-Value	HRPD (*N* = 31)
FR distance (cm)	24.6 (4.6)	30.7 (5.87)	**0.03**	29.3 (6.1)
Sway area (mm^2^)	20.3 (36.8)	14.5 (13.5)	0.50	10.3 (14.6)
Velocity AP (mm/s)	21.8 (30.3)	18.9 (14.6)	0.78	25.0 (21.3)
Velocity ML (mm/s)	22.4 (24.7)	17.2 (12.8)	0.50	17.6 (17.0)
Acceleration AP (mG)	455 (189)	582 (146)	**0.04**	627 (169)
Acceleration ML (mG)	37 (19)	66 (39)	**0.02**	55 (43)
JERK AP (mG/s)	4.6 (6.3)	4.5 (4.2)	0.97	18.1 (40.2)
JERK ML (mG/s)	9.4 (12.7)	5.8 (7.0)	0.38	9.9 (11.0)
MPF (Hz)	6.1 (0.5)	5.5 (0.5)	0.40	6.0 (0.3)

*Data are presented with mean (SD). Values of PD patients and controls were compared using Student’s *t*-test. HRPD values are only included here for comparison purposes. Note the high JERK values in particular in the AP direction of the HRPD individuals, compared to PD and controls. The relevant parameters for the model are marked bold*.

*AP, anterior–posterior; FR, functional reach; ML, mediolateral; MPF, mean power frequency*.

### Model-based approach to differentiate HRPD from controls

The three above-mentioned parameters that differed significantly between PD patients and CO were included in a model to test their utility to differentiate HRPD from CO. The inclusion of these parameters yielded an AUC of 0.70, with a specificity of 70%, and a sensitivity of 77%. The additional inclusion of the JERK parameters in the AP and ML direction improved the AUC to 0.77 and the specificity to 85%, without relevantly affecting sensitivity (74%). AUC as well as specificity and sensitivity values of different models were calculated (Table 3).

**Table 3:** **Area under the curve (AUC), sensitivity, and specificity, as well as *r*^2^ of combinations of parameters, which have been found to be significantly different between patients with Parkinson’s disease (PD) and controls, and which have previously shown to be altered in individuals with high risk for future PD (HRPD) (Maetzler et al., 2012), for the discrimination of HRPD from controls**.

	AUC	Sensitivity (%)	Specificity (%)	*r*^2^ (%)
FR	0.51	41	75	0.6
A AP	0.56	55	66	1
A ML	0.61	70	58	1
FR + A AP	0.60	77	66	3
FR + A ML	0.63	51	83	3
FR + A AP + A ML	0.70	77	70	5
JERK AP	0.61	48	85	3
JERK ML	0.61	48	86	3
A AP + A ML + JERK AP + JERK ML	0.63	35	93	4
FR + A AP + A ML + JERK AP + JERK ML	0.77	74	85	10

*A AP, acceleration in anterior–posterior direction; A ML, acceleration in mediolateral direction; FR, functional reach*.

## Discussion

The main finding of this study is that a combination of markers extracted out of an iFR assessment differentiates HRPD from CO with fair accuracy, sensitivity, and specificity. Our observations basically confirm findings from previous studies investigating (subtle) motor deficits in HRPD individuals (Gaenslen et al., 2011; Mirelman et al., 2011; Maetzler and Hausdorff, 2012; Maetzler et al., 2012; Postuma et al., 2012a). The results support the idea that challenging motor tasks may have a particularly high potential to discover those individuals who eventually convert to PD.

The three parameters of the iFR, which separated PD from CO and also reached a satisfactory discrimination between HRPD and CO, fit well with the currently existing biomechanical picture(s) of PD. As discussed in the Section “Introduction” (Smithson et al., 1998; Mancini et al., 2008) and also shown in this study, PD patients yielded shorter FR distances than did age-matched CO. This is in agreement with the previously described reduced maximum balance range of PD patients detectable even in early PD stages (Horak et al., 2005; Menant et al., 2011). The reduced mean AP and ML accelerations observed during the FR in PD patients compared to CO may be best explained by the following symptoms/reasons. First, PD patients suffer from an increased muscle tone and hypokinesia, leading to reduced compensatory motor response. Second, reduced acceleration in the AP direction of the PD patients compared to CO may also be due to a difference of general sway strategy. Healthy older adults prefer an ankle strategy, which mainly influences parameters in the AP direction (Runge et al., 1999; Horak et al., 2005; Colnat-Coulbois et al., 2011), whereas PD patients rather prefer a hip strategy, which has lower influence on AP parameters (Horak et al., 2005). Moreover, the reduced AP acceleration observed in the PD patients may – at least partly – be explained by the known undershooting of reaching to targets typically associated with PD (Demirci et al., 1997).

We found that a panel of parameters of the iFR separated HRPD better from CO than any single parameter. This observation suggests that not a single parameter but rather a network including a number of associated parameters is affected in the HRPD individuals (Maetzler et al., 2013). From a “biomarker” point of view, the consideration of a panel of parameters rather than a single parameter within a network may increase the usefulness of a model to delineate individuals of interest. This has been suggested and investigated in studies differentiating PD from CO using biomechanical (van der Kooij et al., 2007; Zijlstra et al., 2012; Maetzler et al., 2013; Schoneburg et al., 2013) and biochemical approaches (Bogdanov et al., 2008; Morgan et al., 2010; Farooqui and Farooqui, 2011; Shi et al., 2011; Mielke et al., 2013; Reeve et al., 2013; Subramaniam and Chesselet, 2013; Mielke and Maetzler, 2014; Park et al., 2014). The most-often mentioned advantage of such model-based approaches is the consideration of compensation mechanisms, which certainly play an important role in chronic and progressive diseases such as PD (Maetzler et al., 2013). In our particular situation investigating HRPD individuals with a motivation-dependent task, the model-based approach has an additional advantage: this approach can account for different strategies to perform the task. For example, if a HRPD individual is highly motivated and choses to reach as far as possible, the FR distance may be control-like, however, correction mechanisms will be maximally challenged. This will be reflected by changes in the acceleration (and JERK) parameters included in the model. If the individual decides to take a low risk to fail, the FR distance will be PD-like, however, the acceleration parameters will not be specifically altered. In this particular study, a model considering the parameters relevant for such a scenario enabled us to approach a very good specificity.

A further important observation of this study is that consideration of *U*-shaped progress of certain balance parameters as previously suggested for a static balance paradigm (Maetzler et al., 2012) increases the accuracy to differentiate HRPD individuals from CO also when testing the limit of stability (i.e., JERK parameters, see Figure 2). Ultimately, by combining quantitative FR parameters, which show either a linear, or a non-linear *U*-shaped or inversed change from normal to PD, our model yielded a fair accuracy, specificity, and sensitivity to differentiate HRPD from CO.

The study faces some limitations. First, it used a cross-sectional design and did not (yet) validate its findings by inclusion of PD converters. A further limitation of the method is that, although AUC values are fair in differentiating HRPD from CO, the combination of parameters from the iFR explain only a minority of the difference between the groups (Table 3).

However, we follow the study participants longitudinally and will thus have the opportunity to test our results in the future. We feel that these cross-sectional data are still an important contribution to the field, because they may justify the inclusion of this relatively simple task in ongoing studies on prodromal PD. Second, as no perfect definition of HRPD individuals exist to date, it is probable that not all of our HRPD will eventually develop PD. However, our inclusion criteria considered the increasing risk with increasing numbers of risk and prodromal factors (Liepelt et al., 2011; Ross et al., 2012; Siderowf et al., 2012), which is most probably one of the best models for the definition of such a cohort currently available. Third, it is not fully investigated yet to which extent the reduced limits of stability in PD are rather a compensatory mechanism (Demirci et al., 1997; Maetzler et al., 2013) or have an underlying pathophysiology related to postural instability (van Wegen et al., 2001; Błaszczyk et al., 2007; Mancini et al., 2012; Schoneburg et al., 2013). It could be that the underlying mechanisms of degeneration and compensation are different in HRPD and PD. However, “clinical PD” must be considered as the best endpoint for investigations of prodromal PD phases currently available (Siderowf and Stern, 2008; Gaenslen et al., 2011; Berg and Bandmann, 2013; Berg et al., 2013). Moreover, as changes in the prodromal, or from the prodromal to the clinical phase may not always be linear (Siderowf and Stern, 2008; Maetzler and Hausdorff, 2012), we included parameters in our (second) model, which have been shown to be altered in HRPD (but not in PD, compared to CO) in a previous study investigating static sway under challenging conditions.

Fourth, the particular experimental setting has not been validated yet. However, Mancini et al. (2012) have shown in early PD patients and healthy older adults that trunk accelerometry parameters during quiet stance are strongly associated with balance platform parameters. Thus, experiments with accelerometry-based quantitative sensors are a useful approach for measuring parameters at (or nearby) the center of mass during quiet stance (e.g., Moe-Nilssen and Helbostad, 2002; Lamoth et al., 2009; Lindemann et al., 2012). As our approach is basically comparable to a quiet stance experiment, we argue that the quantitative data obtained in this experiment reflects a kind of sway behavior during quiet standing. However, a direct validation experiment has not been performed. Fifth, the FR test itself faces some limitations: it is not related to center of mass (CoM) or center of pressure (CoP) limits of stability. It is performed only in one direction and does not allow an identification of the type of balance problem (Mancini and Horak, 2010). Still it has been associated with center of pressure excursion (COPE), and is related to the margins of stability and a functional assessment of an essential everyday life task (Duncan et al., 1990).

## Conclusion

The approach presented here does not definitely allow differentiating between degeneration and compensation aspects of balance at the limit of stability in PD and HRPD. Still, we believe that it can relevantly contribute to an assessment panel for definition of HRPD in future studies. In combination with tasks that assess other motor as well as non-motor domains of the PD spectrum, the iFR could serve as an important contribution to an assessment battery that yields an acceptable positive predictive value for future PD.

## Author Contributions

Sandra E. Hasmann, Daniela Berg, Inga Liepelt-Scarfone, and Walter Maetzler made substantial contributions to the acquisition, analysis, and interpretation of data for the work. Markus A. Hobert, David Weiss, Ulrich Lindemann, and Johannes Streffer made substantial contributions to the acquisition of the data. Sandra E. Hasmann and Walter Maetzler drafted the paper, all remaining authors revised the draft critically for important intellectual content. All authors gave their final approval of the version to be published, and agree to be accountable for all aspects of the work in ensuring that questions related to the accuracy or integrity of any part of the work are appropriately investigated and resolved.

## Conflict of Interest Statement

There are no conflicts to declare. Johannes Streffer is employed by Johnson and Johnson, which sponsored the PMPP study. The funding of the PMPP study is pre-competitive.

## Supplementary Material

The Supplementary Material for this article can be found online at http://www.frontiersin.org/Journal/10.3389/fnagi.2014.00286/abstract
