# Supplementary material for: Instrumented Functional Reach Test Differentiates Individuals at High Risk for Parkinson’s Disease from Controls
Source: Front Aging Neurosci. 2014 Oct 24;6:286. doi: 10.3389/fnagi.2014.00286 (PMC4208400; doi:10.3389/fnagi.2014.00286)
Supplement: Supplementary file 1 [file Table_1.DOCX]

| **Parameter** | **Extraction method** | **Unit** | **Description of parameter** |
| --- | --- | --- | --- |
| **Sway Area** | semiautomatic | mm²/s | Area of an ellipse containings 95% of the data points (red ellipse). The area is divided by the duration of the measurement [seconds]. 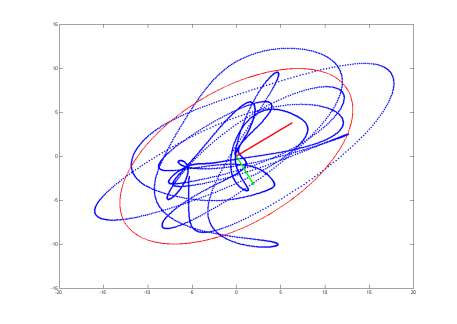 |
| **Velocity** | semiautomatic | mm/s | … is the integration of the acceleration signal.  **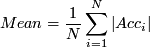** |
| **Acceleration** | semiautomatic | mG/s | … is the vector of the acceleration in the transverse plane. 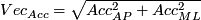 |
| **JERK** | semiautomatic | mG/s | … is the rate of change of the acceleration.  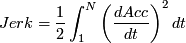 |
| **MPF** | semiautomatic | Hz | … is a measure for the frequency content of the signal.   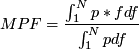 |

**Supplementary Table 1.** Extracted parameters out of the sway raw data, presented with extraction method, unit as well as a description of the parameter. MPF, mean power frequency.
